# Network-level restoration of impaired memory function in early Alzheimer’s disease via GluN2B modulation: insights from a hippocampal CA1–CA3 model

**DOI:** 10.3389/fncom.2026.1953352

**Published:** 2026-10-01

**Authors:** Fabio Librizzi, Saana Seppälä, Marja-Leena Linne, Michele Migliore, Ausra Saudargiene

**Affiliations:** 1 Istituto di Biofisica, Consiglio Nazionale delle Ricerche, Palermo, Italy; 2 Faculty of Medicine and Health Technology, Tampere University, Tampere, Finland; 3 Neuroscience Institute, Lithuanian University of Health Sciences, Kaunas, Lithuania; 4 Department of Informatics, Vytautas Magnus University, Kaunas, Lithuania

**Keywords:** Alzheimer’s disease, amyloid beta, CA1 microcircuit, computational network, memory storage/recall, NMDAR

## Abstract

Memory storage and recall are essential tasks for the healthy function of the hippocampus, adversely affected by neurodegenerative diseases, such as Alzheimer’s Disease (AD). Pathological levels of amyloid-β peptide (Aβ), found already in early-stage AD, can disrupt the NMDAR-dependent plasticity at hippocampal networks. Here we investigate these effects in a biophysically detailed computational model of a hippocampal CA1–CA3 network and examine potential mechanisms of functional recovery. We show that, at a network level, long-term potentiation (LTP) impairments caused by Aβ accumulation can be substantially restored by partially blocking the GluN2B–NMDA receptors—a mechanism previously observed and modeled only at a single cell level. Experimental access to network-level processes of memory storage and recall in pathological conditions remains highly challenging. Therefore, computational modeling may help to bridge the gap between cellular impairments and system-level cognitive dysfunction. Our work transfers the established GluN2B-dependent rescue mechanisms from single cells to a network-level system of hippocampal pyramidal neurons and interneurons. The results demonstrate that also at a higher cognition level, the partial inhibition of the activity of GluN2B–NMDA receptors can be an effective strategy to counteract early AD impairments on memory storage and recall.

## 1 Introduction

Memory storage and recall are fundamental processes in which the hippocampus plays a pivotal role. Long-term synaptic plasticity is widely recognized as a key process underlying these functions (Malenka and Bear, 2004; Malenka and Nicoll, 1999). Synaptic plasticity mechanisms such as long-term potentiation (LTP), long-term depression (LTD) and spike-timing-dependent synaptic plasticity (STDP) have been detected in the CA3 Schaffer collateral-CA1 pyramidal neuron synapses within the hippocampal CA1 area (Bliss and Collingridge, 1993; Malenka and Nicoll, 1993; Dudek and Bear, 1992). Memory-related neurogenerative diseases, particularly Alzheimer’s Disease (AD), adversely affect the hippocampal brain region, leading to memory impairments already at early stages of the disease. Especially in the hippocampal CA1 area, the pathological mechanisms behind AD impair LTP and LTD, compromising memory storage and recall (Abramov et al., 2009; Pousinha et al., 2017; Opazo et al., 2018; Pousinha et al., 2019).

The induction of STDP, LTP and LTD requires the activation of the N-methyl-D-aspartate receptors (NMDARs) (Bliss and Collingridge, 1993; France et al., 2017), which are essential modulators for long-term synaptic plasticity changes. Underlying the impairment of the synaptic plasticity mechanisms, alterations in the processing and clearance of the amyloid precursor protein (APP) have been observed in early-stage AD (O’Brien and Wong, 2011). Such deviations can lead, for example, to accumulated levels of APP fragments like Amyloid-β peptide (Aβ). Aβ, which is known to inhibit hippocampal LTP *in vivo* (Walsh et al., 2002), has been found to bind to the NMDARs, potentially causing excess activation of the target site, eventually leading to neurodegeneration (Wang and Reddy, 2017; Peng et al., 2022). In general, overactivation of NMDA receptors may drive neuronal damage and dysfunction, see, e.g., Zhang et al. (2025). As a consequence, their inhibition can be a potential strategy in the treatment of different disorders. In fact, several compounds acting as antagonists for NMDA receptors have been shown to be useful in conditions like depression (Khabir et al., 2022), epilepsy (Chen et al., 2022), and neurodegeneration (Reisberg et al., 2003; Matsunaga et al., 2015; Rascol et al., 2021).

In recent studies, the role of impaired function of a specific NMDAR subunit, GluN2B–NMDA receptor, has been studied in the AD pathology (Opazo et al., 2018; Dainauskas et al., 2023a,b). The GluN2B–NMDA receptor subunit activity has a critical role for the induction of LTP and LTD in CA1 pyramidal neurons (Shipton and Paulsen, 2014), thus affecting synaptic plasticity processes in the hippocampal area (Gardoni et al., 2009; Brigman et al., 2010). GluN2B–NMDARs interact with and modulate the calcium/calmodulin kinase II (CaMKII), which is recognized as an essential protein for the induction of LTP by increasing the ratio of active α-amino-3-hydroxy-5-methyl-4-isoxazolepropionic acid receptors (AMPARs) at the synapse. Oligomeric Aβ can lead to overexpression of the CaMKII protein, tightly associated with the GluN2B–NMDAR activity (Opazo et al., 2018). The main properties of the CA3–CA1 synapse, including the interactions between the relevant molecules, receptors, and Aβ, are illustrated in Figure 1.

**Figure 1:**
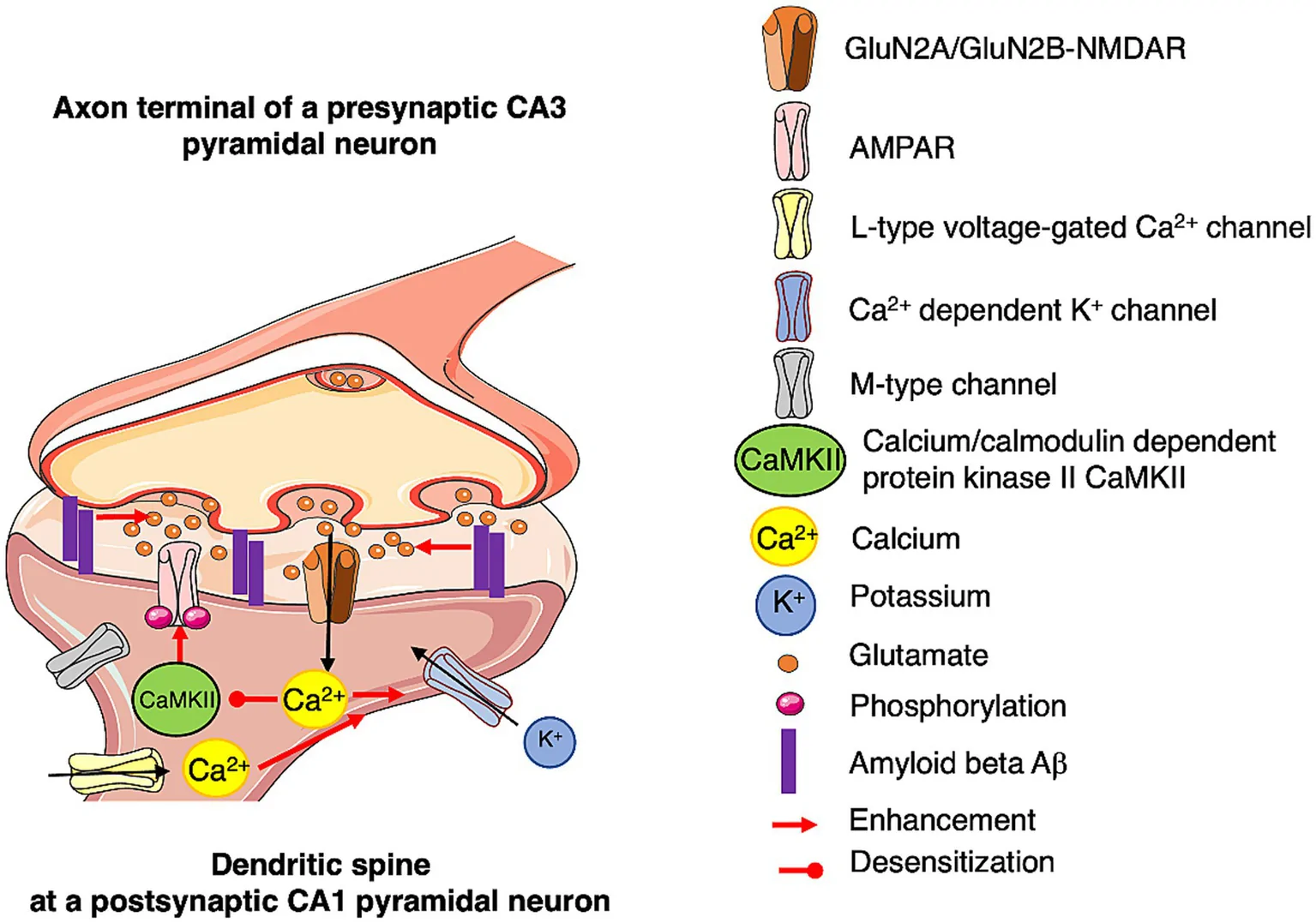
Schematic illustration of the CA3–CA1 synapse model and its key properties under healthy and Alzheimer’s disease (AD) conditions. The figure shows the axon terminal of a presynaptic hippocampal CA3 pyramidal neuron and a dendritic spine of a postsynaptic CA1 pyramidal neuron. In the AD condition, increased Aβ alters the properties of GluN2A- and GluN2B-containing NMDA receptors (NMDARs) at CA3–CA1 synapses, thereby affecting the LTP/LTD mechanisms underlying synaptic plasticity. The overall network architecture is identical across the control, AD, and rescue conditions.

In pathological conditions, such as early AD, the inhibition of the overly activated GluN2B subunit may enable the restoration of the impaired LTP in CA1–CA3 synapses. In fact, this has been demonstrated experimentally with the pharmacological blockage of the GluN2B–NMDA receptor, by means of a specific subunit antagonist, ifenprodil (Opazo et al., 2018; Rönicke et al., 2011).

Computational investigations at the single‑neuron level have further elucidated the specific role of GluN2B–NMDA receptor in the plasticity of CA3–CA1 synapses, both in normal and pathological AD-like conditions, shedding light on the biophysical mechanisms underlying the above mentioned GluN2B-mediated recovery (Dainauskas et al., 2023a,b). Whether such mechanisms remain effective when synaptic impairments are distributed across a neuronal population, and how they ultimately affect memory storage and recall at a network level, cannot be readily addressed experimentally. Computational network models therefore may play an important role in extending cellular‑level insights to higher cognitive levels.

Importantly, the synaptic plasticity model employed here is grounded in previous experimental and computational works (Opazo et al., 2018; Pousinha et al., 2017; Dainauskas et al., 2023a,b). The model and the AD-related modifications incorporated in the CA3–CA1 synapse were previously constrained and validated against electrophysiological observations of Aβ-induced and GluN2B-mediated alterations in hippocampal synaptic plasticity and CA1 neuron function. In particular, previous work demonstrated that the model reproduces experimentally observed impairments in LTP and their rescue following partial GluN2B antagonism (Dainauskas et al., 2023b). The objective of the present study is therefore not to re-establish these cellular mechanisms, but rather to investigate whether the same experimentally supported mechanisms remain sufficient to explain alterations in memory storage and recall when embedded into a hippocampal CA1 microcircuit. Consequently, here we extend previous single-cell modeling work (Dainauskas et al., 2023a,b) to a suitable hippocampal CA1–CA3 network (Bianchi et al., 2014), investigating whether experimentally validated Aβ- and GluN2B-dependent mechanisms of synaptic dysfunction and recovery remain effective at the level of memory storage and recall in a neuronal population. Specifically, the aim is to study the effects of increased Aβ concentration on the memory storage and recall ability of a network of CA1 neurons, describing phenomenologically the effects of early AD and eventually to investigate the mechanisms of memory restoration following the blockage of GluN2B–NMDA receptors in a population of neurons. To the best of our knowledge, this is the first study to explore at a system-level the experimentally established phenomenon that impaired synapses can be restored through pharmacological blockade of specific NMDAR subunit. The difficulty of experimentally assessing such phenomena at a network level highlights the importance of exploring the link between single cells dysfunction and higher cognition network level failures in model systems, to further understand memory storage and recall in health and disease.

## 2 Methods

Our computational model framework is based on the network architecture of 100 hippocampal pyramidal CA1 neurons with inhibitory interneurons by Bianchi et al. (2014) (ModelDB acc. no. 151126). To study the role of the GluN2B–NMDA receptor subunit for the recovery of pathological synapses in CA1–CA3 network, we embedded the synaptic plasticity model by Dainauskas et al. (2023a,b) to the CA1–CA3 network.

### 2.1 System configuration

The simulations were conducted with NEURON model builder Hines (1997) (version 8.2.6 or 8.2.4), with MPI (Message Passing Interface) using up to 48 cores on Cineca G100 and on a local CPU cluster at IBF-CNR, Palermo.

### 2.2 CA1–CA3 network architecture

We used a CA1 network reconstruction (Bianchi et al., 2014) to describe the properties of memory storage and recall at the network level. The architecture—see Figure 1 in Bianchi et al. (2014) for a schematic representation—comprises 100 hippocampal CA1 pyramidal neurons with four types of inhibitory interneurons—oriens-lacunosum moleculare (iOLM), basket (iB), bistratified (iBS), and axo-axonic (iAA) cells—influencing the input–output properties of the CA1 pyramidal neurons and the mechanisms for memory encoding. For each simulation, 20 CA1 neurons were randomly selected within the 100 neurons. For modeling the interneurons and the morphology of the CA1 neurons, the work by Cutsuridis et al. (2010) was used. Each CA1 neuron was modeled with 15 compartments, with a modified ionic current distribution from Bianchi et al. (2012). The compartments of each CA1 neuron consisted of soma, axon, oriens proximal and distal compartments of the basal dendrites, radiatum proximal, medial and distal compartments of the apical trunk, and lacunosus moleculare, thin, medium and thick compartments. Currents were distributed in the compartments of each CA1 cell according to Bianchi et al. (2014).

The network has excitatory inputs from EC (20 inputs) and CA3 Schaffer collaterals (100 inputs), and inhibitory inputs from the septum (10 inputs). As in the original network architecture (Bianchi et al., 2014), the excitatory inputs from EC and CA3 Schaffer collaterals represent the preprocessed sensory information received from the neocortex, providing contextual input from the internal memory. The EC and CA3 inputs are activated by gamma bursts (30–80 Hz), coupled with a theta rhythm (4–8 Hz; Fuentemilla et al., 2010; Lisman, 2010). Each excitatory CA3 input creates two synaptic connections on the proximal dendrites of each CA1 neuron, comprising one AMPA-mediated and one NMDA-mediated synapse. Each excitatory EC input precedes the CA3 input by 9 ms and forms two synaptic connections on the distal dendrites of 20 randomly chosen CA1 neurons, utilizing AMPA-mediated synapses without implementation of synaptic plasticity. Further, the inhibitory septal inputs are modeled as bursts of synaptic activations, occurring at a mean frequency of 50 Hz, with a duration of one-third of a theta cycle.

### 2.3 Synaptic plasticity model with increased Aβ levels

To study the role of GluN2B–NMDA receptor in the memory storage and learning, we applied a recently published phenomenological NMDAR-based plasticity model (Dainauskas et al., 2023a,b). The model is built on the assumption that LTP is highly dependent on the GluN2B–NMDAR subunit, whereas LTD is mediated by the GluN2A-NMDAR subunit. The LTP and LTD functions are voltage-dependent and contribute to the change in the weight of the AMPA receptor. The synaptic weights evolve according to the STDP rule, based on the relative timing of the pre- and postsynaptic spikes. The plasticity model is restricted to the CA3–CA1 synapses; thus, the plasticity was implemented only for the inputs between CA3 and CA1, by initializing all 100 synaptic connections separately.

The CA3–CA1 coupling represented in the model is chemical excitatory synaptic transmission through Schaffer collateral inputs (electrical coupling is not included). Presynaptic CA3 activity activates postsynaptic AMPA and NMDA receptors on the CA1 pyramidal neuron. The NMDA component is further separated into GluN2A- and GluN2B-containing receptor contributions, which mediate the LTD- and LTP-related plasticity terms, respectively. Synaptic efficacy is represented by the AMPAR synaptic weight, which is dynamically modified according to the voltage- and NMDAR-dependent plasticity rule. The equations can be found in Dainauskas et al. (2023a).

All plasticity parameters were taken directly from Dainauskas et al. (2023b), with the exception of the midpoint values for the Hill functions describing the activation of the LTP and LTD components (see Dainauskas et al., 2023a for a detailed description). These two parameters regulate the interplay between LTP and LTD, and thus the balance between potentiation and depression of the synaptic weights; as such, they require a specific adaptation to the system under study. For our network, they were set to 0.72 and 1.80 pS/cm^2^, respectively, in order to have a proper potentiation profile, in a suitable time window, during memory storage under control conditions. Other synaptic connections are modeled as in Bianchi et al. (2014).

To describe the molecular pathology in early AD, we modeled the impaired neuronal activity due to augmented levels of Aβ by decreasing the synaptic density to 80% (Opazo et al., 2018), decreased excitatory neurotransmission by increasing free glutamate concentration to 120% (Abramov et al., 2009), and impaired LTP by modifying the shape of the GluN2B–NMDAR-dependent LTP function to abolish LTP even when the GluN2B–NMDAR subunit is activated (Dainauskas et al., 2023b). In particular, the midpoint value of the LTP inhibition component was set to 1.66 pS/cm^2^. The explicit mathematical implementation of decreased synaptic density, increased free glutamate concentration and LTP modulation can be found in Dainauskas et al. (2023b), Supplementary material.

To demonstrate the recovery resulted by the partial blockage of the GluN2B–NMDAR subunit, we decreased the conductivity of this specific unit in the model, while preserving the previously explained changes to describe the effects of the accumulation of Aβ. These parameter modifications are in line with the previous work at single neuron level by Dainauskas et al. (2023b).

### 2.4 Simulation protocol for memory storage and recall

The principles of memory storage and recall were implemented as in Bianchi et al. (2014), originating from the work by Hasselmo et al. (2002), Cutsuridis et al. (2010) and Lisman (2010). In brief, the each CA1 receives input from EC and CA3, arriving at different phase of the theta cycle. At one half of a theta cycle, a strong input from the EC allows the encoding of new patterns. During the other half, input from the CA3 allows the network to recall the stored memory pattern in the absence of the EC input. Adapting this model, the storage and recall functions were modeled under three different conditions for 100 theta cycles: control, accumulated Aβ describing early AD, and recovery of affected synapses.

During the storage phase, both EC and CA3 are active. The EC inputs activate the iB and iAA interneurons, as well as 20 randomly selected CA1 neurons from the network. Additionally, CA3 inputs activate iAA, iB, iBs and all CA1 neurons. As stated, the EC and CA3 inputs arrive with a 9 ms difference. If the total effect of the inputs leads to strong enough activity, LTP is induced.

During the recall phase, only CA3 inputs are possible. The network receives inhibitory inputs from the septum, and all the interneurons are inhibited. CA1 escape the inhibition and activate the iOLM. However, the CA1 distal dendrites are inhibited, thus there are no inputs from the EC. Also, CA3 are initially inhibited, except if they have a connection to those CA1 neurons that are potentiated during recall, meaning neurons that belong to the right memory pattern.

Different sets of patterns are presented to the network model, in order to evaluate how well the network recalls the stored memories under different conditions. The presented patterns can be either non-orthogonal or orthogonal, activating either overlapping (non-orthogonal patterns) or non-overlapping (orthogonal patterns) set of synapses, respectively. The patterns are presented as binary vectors. Since the network model has 100 CA1 cells and uses 20-input patterns, the binary vectors have a length of *N* = 100 with nP = 20 active units, meaning that a maximum of five orthogonal patterns (*N* = 100/nP = 20) can be presented to the network. For non-orthogonal patterns, the overlap between patterns is permitted and their number is not bounded by the *N*/nP limit that applies to the orthogonal case.

The recall quality, describing how well the network succeeded to retrieve the previously stored patterns was measured as the normalized dot product describing the correlation of the input and output patterns, as described in Bianchi et al. (2014):

$$Q(P^{\prime },P)=\frac{\sum_{i=1}^{N}P_{i}*P_{j}^{'}}{\sqrt{\sum_{i=1}^{N}P_{i}*\sum_{j=1}^{N}P_{j}^{'}}}$$ (1)

where *P'* and *P* represent the output and input patterns, respectively, and *N* is the total number of the CA1 neurons. For the normalized recall quality, value 1 indicates perfect identity between input and output patterns. The value clearly decreases when input and output patterns differ. For our system (20 inputs for each pattern of 100 CA1 neurons), a pattern can be considered satisfactorily recalled when the value exceeds a threshold of 0.447, corresponding to the case in which all neurons in the recall pattern are active. In fact, in our network, each memory is represented by a specific pattern of (nP = 20) active neurons among the (*N* = 100) CA1 neurons. Successful recall therefore requires the 20 pattern neurons to be active while the remaining 80 neurons remain silent. Recall quality is quantified by comparing the neurons that are active during recall with those belonging to the target pattern. Recall quality decreases both when pattern neurons fail to activate and when neurons outside the target pattern become active. We consider a pattern to be satisfactorily recalled when the recall quality exceeds the threshold of 0.447. This threshold corresponds to the recall quality obtained when all 100 CA1 neurons are active. Although this state contains all 20 neurons of the target pattern, it provides no information about which memory was retrieved, because the same fully active output would be produced for every cue.

## 3 Results

We modeled the memory storage and recall ability of a hippocampal CA1–CA3 network under normal and pathological AD conditions and investigated the potential recovery of the affected synapses by means of a partial inhibition of GluN2B function. The first step included the optimization of the parameters of the single synapse plasticity model (Dainauskas et al., 2023a) to adapt it to our CA1–CA3 network. In particular, the values of all the parameters were set according to the single synapse model (Dainauskas et al., 2023a), with the exception of the midpoints of the Hill functions relative to the LTP and LTD components, which were regulated in order to have a suitable synaptic weight potentiation, describing a control condition, when single patterns are presented to the network for storage (Figure 2a; see also Methods). Then, according to previous work (Abramov et al., 2009; Opazo et al., 2018), Aβ accumulation was simulated by increasing glutamate concentration to 120% (Abramov et al., 2009), reducing synapse density to 80% (Opazo et al., 2018) and properly setting the conductance value to produce half activation of the LTP component inhibition in the synaptic plasticity. These adjustments describe the decreased synaptic function due to increased concentration of Aβ, resulting in a strong inhibition of LTP and in the lack of weight increase upon storage protocol (Figure 2b). Reduction of GluN2B function to 40% of its original value produces a recovery of about 90% of the original weight increase during the 25 s of storage protocol (Figure 2c). The recovery fraction strongly depends on the GluN2B attenuation factor, as shown in the inset in Figure 2c, with 0.4 µS providing the maximum recovery for our network. To assess whether this recovery is specific to the GluN2B-dependent mechanism, rather than reflecting a nonspecific reduction of excitatory drive, we performed additional control simulations in which AMPA receptor conductance, instead of GluN2B–NMDAR conductance, was reduced across a range of values (from 20% to 80% of its original value) under the same Aβ-affected conditions (data not shown). None of these non-specific reductions produced any recovery of the synaptic weight increase during storage, indicating the specificity of the GluN2B–NMDAR dependent mechanism.

**Figure 2:**
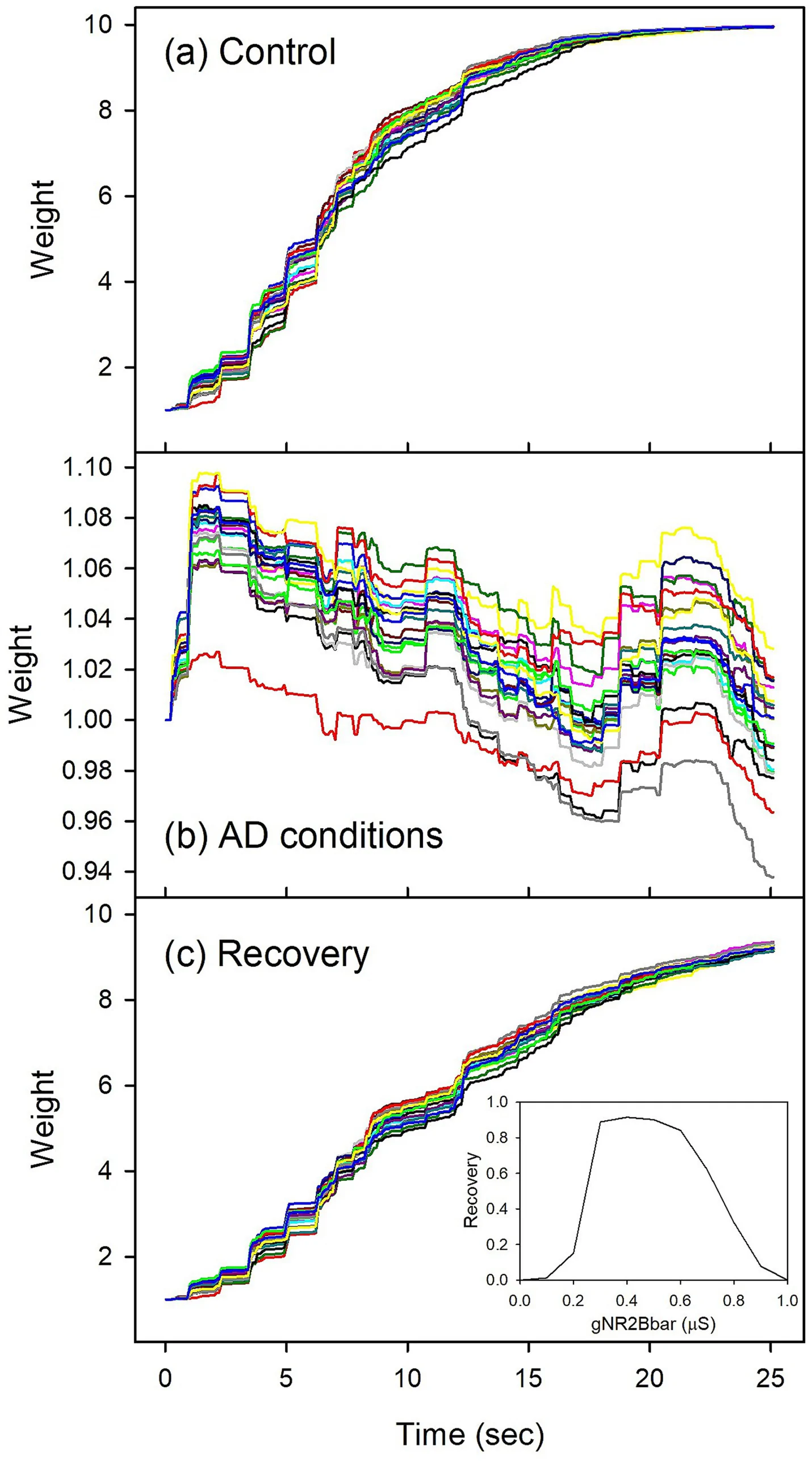
Synaptic weight dynamics during pattern storage and recall. Time course of the synaptic weights of the 20 CA3 active synapses targeting one of the CA1 neurons involved in the storage of one pattern (100theta cycles of storage and recall); **(a)** control conditions; **(b)** AD conditions with increased Aβ; **(c)** rescue conditions with inhibition of GluN2B function to 40% of its initial value. The inset in panel **(c)** shows the rescue fraction as a function of GluN2B reduction. Under AD conditions, the accumulation of synaptic potentiation is impaired, resulting in reduced synaptic strengthening compared with the control condition. The introduced rescue mechanism largely restores synaptic potentiation, indicating that the synaptic deficit is reversible within the model.

Once the parameters were optimized for single pattern storage and recall, we investigated the network performance in the storage and retrieval of multiple patterns, both orthogonal and non-orthogonal. The pattern separation performed by the hippocampus allows the memories to be stored as non-overlapping and unique events; for review see, e.g., Yassa and Stark (2011) and Leal and Yassa (2018). In computational models, orthogonal patterns can be used to demonstrate the formation of such unique memories. Since the network comprises 100 CA1 cells and uses 20-input patterns, a maximum of five orthogonal patterns (100/20) can be presented to it. There is no limit for the number of non-orthogonal patterns, due to their organization. Figure 3 shows the network’s response in healthy conditions, during the recall of a single pattern (2.5 s, 10 cycles) after the storage (25 s, 100 cycles) of five orthogonal patterns (left panels), five non-orthogonal patterns (center) and 50 non-orthogonal patterns (right). In particular, Figure 3 shows input cells spikes (top), pyramidal cells spikes, the recall quality (as defined by Equation 1 in Methods) and the soma voltage for two CA1 neurons, one involved (black line) and one not involved (red line) in the recalled pattern. As expected, the recall is nearly perfect when five orthogonal patterns are introduced, since the patterns are perfectly separated. For non-orthogonal patterns, on average, the quality factor decreases even when only five patterns are stored, mainly due to the spurious spiking of cells not involved in the recalled pattern (Figure 3, lower panels). Recall becomes poor in the case of 50 stored pattern (right panels), approaching the threshold value of 0.45, corresponding to the spiking of all 100 CA1 neurons.

**Figure 3:**
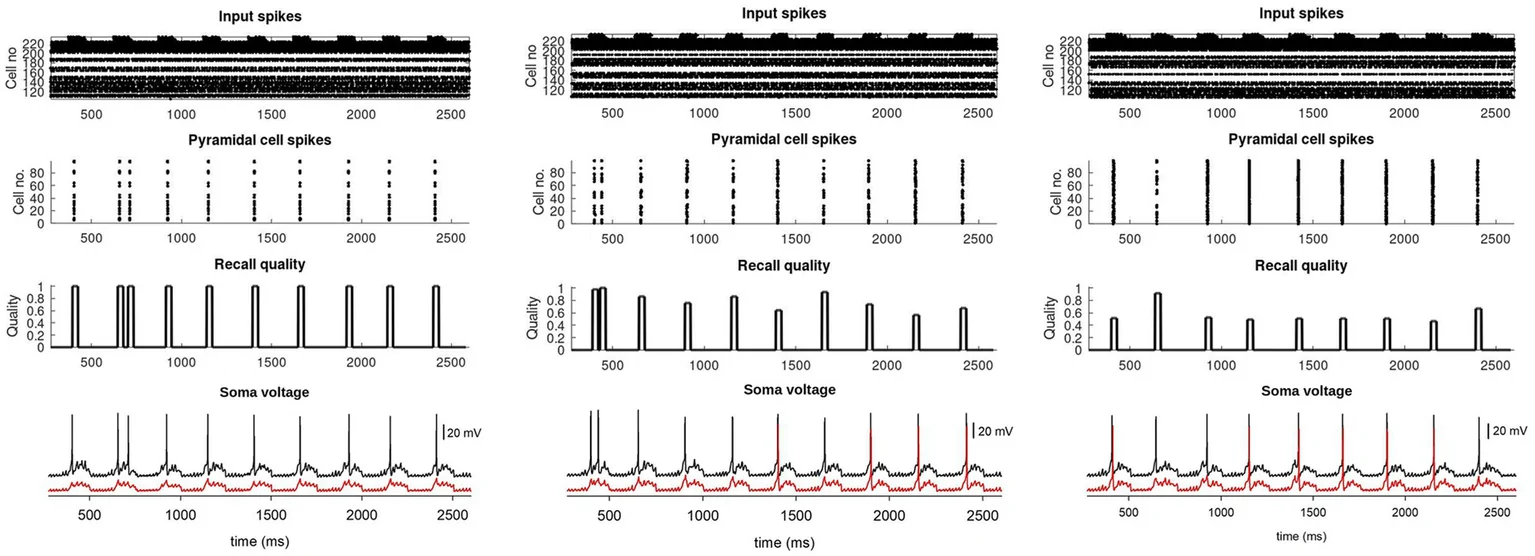
Typical examples of single pattern recall for our network in control conditions after the storage of different combinations of patterns. Left: five orthogonal patterns; center: five non-orthogonal patterns; right: 50 non-orthogonal patterns. From top to bottom panels: raster plot of the spiking times for septum (10 inputs), EC (20 inputs), CA3 (100 inputs) and interneurons (five inputs); pyramidal cell spiking profile; recall quality factor; soma voltage trace for two CA1 neurons, one involved (black) and one not involved (red) in the recalled pattern. The recall quality is always perfect when five orthogonal patterns are stored (left); recall is less efficient in the case of five non-orthogonal patterns (center) and poor for 50 non-orthogonal patterns. The comparison of the response of the network to orthogonal and non-orthogonal patterns in this figure suggests that the network behavior has an essential dependence on the pattern structure.

To evidence the effects on our network of the reduced LTP brought about by the presence of Aβ, and the possible recovery following inhibition of GluN2B activity, the pattern recall ability of the network was monitored with different fractions of AD-affected cells. Specifically, network performances were investigated in the presence of 0%, 20%, 50%, 80%, and 100% of impaired CA3–CA1 synapses, both with orthogonal (Figure 4) and non-orthogonal (Figure 5) patterns, to describe different levels of synaptic impairment during AD. Figure 4a reports the average quality factor in pattern recall as a function of the number of stored orthogonal patterns, for the different fractions of AD-affected synapses and both for original (black) and reduced (red) GluN2B activity (to the optimal value of 0.4 μS). As previously said, with 100 CA1 pyramidal cells and 20-inputs patterns, a maximum of five orthogonal patterns can be used. Figure 4a clearly shows that, as expected, the recall quality does not depend at all on the number of stored patterns since, during storage, orthogonal patterns do not affect each other. On the contrary, the quality decreases significantly when increasing the fraction of AD-affected cells (black curves). This effect is strongly counteracted by the reduction of GluN2B activity to 40% of its original value, up to the point that in rescue conditions the quality factor almost approaches its maximum value even in the case of 100% AD-affected cells (Figure 4a). However, it must be noted that, as shown in Figure 4b, also the average number of network spikes during the recall strongly depends on the fraction of AD-affected cells. When 50% or more of the synapses are impaired by AD, the reduction of GluN2B activity restores in part the average number of spikes (Figure 4b, red). For 0% or 20% of AD-affected cells, the average number of spikes turns to be slightly lower in the case of reduced GluN2B activity, as reasonable; in fact, in the presence of healthy cells the lowering of GluN2B conductance simply results in a less efficient cells response. To take into account the general ability of the network to respond to stimuli, both in terms of number of spikes and quality of the recall, Figure 4c shows the recall quality factor normalized for the average number of network spikes. This parameter appears to be more suitable for a better quantification of network performance in memory recall. As shown in Figure 4c, also for this parameter, reduction of GluN2B activity partly prevents AD-induced impairments in memory recall, but in a less efficient way, if compared with the simple quality factor shown in Figure 4a.

**Figure 4:**
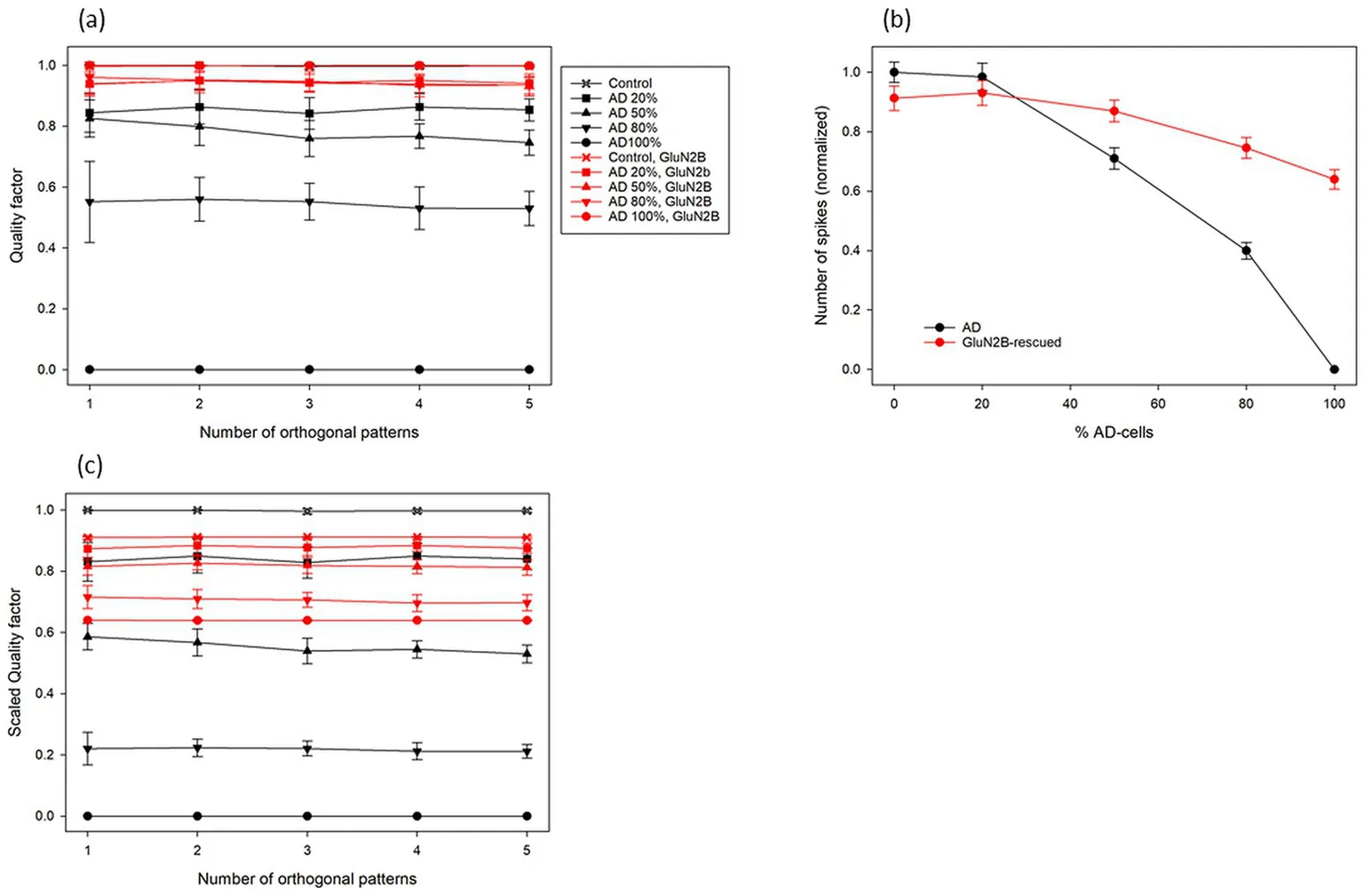
Network behavior during pattern recall with up to five orthogonal patterns stored. Data are presented for different fractions of AD-affected cells (0%, 20%, 50%, 80%, 100%), both for normal (black) and reduced GluN2B activity (to 0.4, red). **(a)** Average recall quality factor as a function of the number of stored orthogonal patterns; **(b)** normalized average number of recall spikes as a function of the fraction of AD-affected cells, for normal and reduced gNR2B activity; **(c)** average recall quality factor as a function of the number of stored orthogonal patterns, scaled for the average number of spikes. For orthogonal patterns, the quality of the recall does not depend on the number of stored patterns. However, it strongly depends on the fraction of AD-affected synapses. Reduction of GluN2B conductance leads to a significant recovery of the average recall quality.

**Figure 5:**
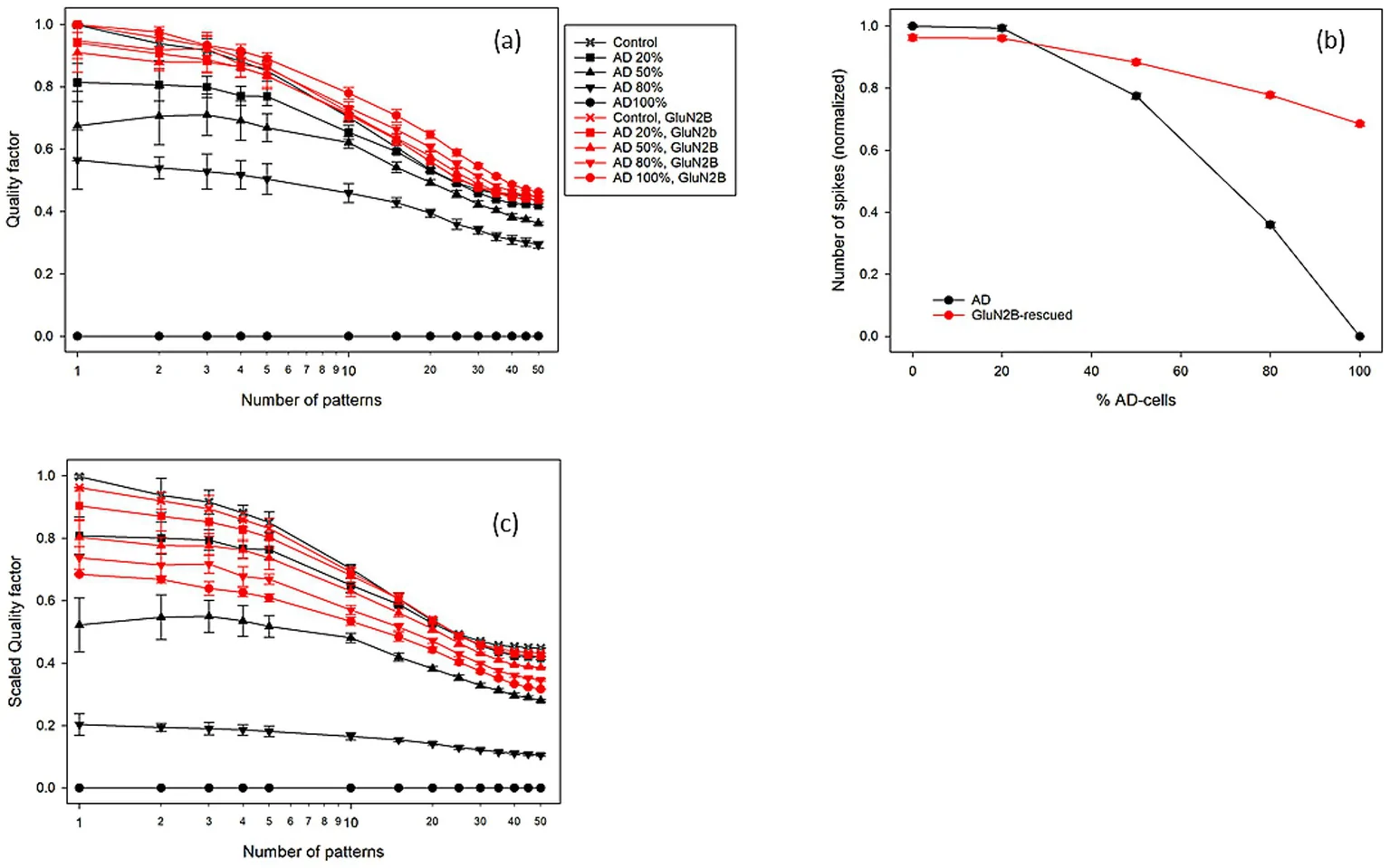
Network behavior during pattern recall with up to 50 non-orthogonal patterns. Data are presented for different fractions of AD-affected cells, for normal and reduced GluN2B (rescued conditions) activity. **(a)** average recall quality factor as a function of the number of stored patterns; **(b)** average number of recall spikes as a function of the fraction of AD-affected cells, for normal and reduced GluN2B activity; **(c)** average recall quality factor as a function of the number of stored patterns, scaled for the average number of spikes. For non-orthogonal patterns, the quality of the recall strongly depends on the number of stored patterns, approaching the threshold value (⁓0.45) when large numbers of patterns are stored, even in healthy conditions. It strongly depends also on the fraction of AD-affected synapses and, as for orthogonal patterns, reduction of GluN2B conductance leads to a significant recovery of the average recall quality.

When non-orthogonal patterns are introduced (Figure 5), we can see a similar trend for recall quality and average number of spikes with different fractions of affected cells and recovery (Figures 5a,b). In addition to this, there is also a clear drop in the quality factor as the number of non-orthogonal patterns increases (Figure 5a). Even in healthy conditions, for large numbers of stored patterns the quality factor approaches the threshold value of ⁓ 0.45, corresponding to the spiking of all 100 CA1 neurons (see Methods). This is quite reasonable, since the CA3–CA1 synapses involved in different non-orthogonal patterns are not distinguished in principle as they are for orthogonal patterns. As a consequence, the storage of a large number of non-orthogonal patterns causes the increase of the conductance weight for essentially all CA3–CA1 synapses, resulting, during the recall phase, in the simultaneous spiking of all CA1 pyramidal cells, whatever pattern is recalled. Figure 5c presents the average quality factor normalized for the average number of recall spikes. Also in this case, as for orthogonal patterns, this parameter appears to be more suitable to measure network performances in memory recall. In fact, the mere quality factor reported in Figure 5a results in some cases better in GluN2B-rescued conditions than in control conditions. This is due to the fact that, as said above, the drop of the quality is due to the spurious spiking of CA3–CA1 synapses not involved in the recalled pattern and that the lowering of current intensity brought about by the partial inhibition of GluN2B may reduce this spurious spiking. It also reduces, however, the average number of spikes and the combination of the two effects leads to the behavior observed in Figure 5c.

## 4 Discussion

Here we modeled the network level outcomes of Aβ-driven AD pathology and GluN2B-mediated recovery by integrating a NMDAR-dependent synaptic plasticity model (Dainauskas et al., 2023a,b) and a network of 100 CA1 pyramidal neurons (Cutsuridis et al., 2010; Bianchi et al., 2014). A key aspect of the present study is that the observed network-level effects emerge from synaptic plasticity mechanisms that were previously constrained and validated against electrophysiological observations at the single-cell level, including Aβ-mediated impairment of LTP and GluN2B-dependent rescue (Abramov et al., 2009; Pousinha et al., 2017; Opazo et al., 2018; Dainauskas et al., 2023b). The present work does not attempt to re-establish these cellular mechanisms. Rather, its novelty lies in assessing whether such experimentally supported processes remain sufficient to account for deficits in memory storage and recall when embedded within a hippocampal CA1 microcircuit.

We studied the changes in the synaptic plasticity and LTP when the cells are exposed to control environment, to conditions mimicking the impairments due to accumulated Aβ in early AD, and to rescue conditions achieved by partial blockage of GluN2B–NMDAR activity on a phenomenological level. Our model presents system-level insights to the relationship of NMDAR-dependent synaptic plasticity and the storage and recall in CA1–CA3 network, specifically the GluN2B-mediated recovery of impaired synapses. Therefore, our results serve as a proof-of-concept that these effects—previously modeled at a cellular level (Dainauskas et al., 2023b)—are reproducible also at a higher cognition level in a population of neurons (Harris et al., 2010; Yang et al., 2018; Gauthier-Umaña et al., 2020).

We successfully implemented the GluN2B-mediated rescue mechanism into the network level interaction of hippocampal neurons. As the GluN2B–NMDAR subunit is essential for synaptic plasticity, the subunit cannot be completely blocked. By reducing the conductivity of GluN2B to 40% of its initial value, we were able to produce optimal recovery for our model, in agreement with experimental results (Rönicke et al., 2011), demonstrating that partial blockade of GluN2B can rescue synaptic function under Aβ pathology. The precise level of GluN2B reduction required for optimal rescue may depend on the specific model configuration and experimental conditions. Therefore, the 40% reduction identified here should not be considered a universal optimum; rather, our main finding is the existence of an intermediate level of GluN2B activity that optimally restores LTP under the conditions studied. Notably, this rescue is specific to the GluN2B-dependent mechanism: control simulations with non-specific AMPA conductance reduction did not produce any recovery, consistent with the non-monotonic dependence of LTP recovery on the degree of GluN2B inhibition (Figure 2c, inset) and with the known role of GluN2B–NMDAR-dependent Ca^2+^/CaMKII signaling in Aβ-induced LTP disruption (Opazo et al., 2018).

The recovered behavior of the network was evaluated against different fractions of affected synapses, both for orthogonal and non-orthogonal patterns to study the network behavior with different level of separation in the memory patterns. The model performed as expected, displaying robust behavior and supporting the presented mechanisms for synaptic recovery in AD-like conditions. In particular, the results indicate that accumulated Aβ disrupts the GluN2B behavior in hippocampal microcircuits, impairing the memory storage and recall especially by affecting the theta-gamma coupling, which can be partly restored by suppressing GluN2B conductivity.

Although characterized at the single-synapse level (Dainauskas et al., 2023b), the observed rescue is not only a consequence of introducing the previously validated GluN2B–NMDAR-dependent plasticity mechanism into the network. The results indicate that the degree of recovery depends on network/population-level variables, such as the fraction of affected CA1 neurons and the structure of the stored patterns (Figures 4, 5). For non-orthogonal patterns, the resulting recall quality depends on how the GluN2B activity reduction affects both the cells included and not included in the recalled pattern. The decrease in the spurious spiking of the cells not involved in the pattern seemed to improve the recall specificity, while the simultaneous decrease in the average spiking can weaken the quality. Even though the prediction of the contribution of the reduced GluN2B activity comes from the local single-synapse mechanisms, the final outcome (e.g., Figure 5c) reflects a network-level interaction between the CA1 neurons.

Inhibition of NMDA receptors has been shown to be an effective therapeutic strategy for a range of conditions (Rascol et al., 2021; Khabir et al., 2022; Chen et al., 2022), including AD, for which unspecific antagonists have been also approved by Food and Drug Administration due to their modest symptomatic effects (Reisberg et al., 2003; Rascol et al., 2021). To our knowledge, no specific antagonist for the GluN2B subunit has been approved as a possible therapeutic agent in AD. Nevertheless, increasing experimental evidence suggests that GluN2B plays a crucial role in Aβ-induced synaptic dysfunction and early cognitive impairment (Pousinha et al., 2017; Rönicke et al., 2011; Hanson et al., 2015; Li et al., 2011). Here we want to stress the importance of further investigating the selective pharmaceutical blockage of the GluN2B subunit by means of specific antagonists such as ifenprodil (Opazo et al., 2018; Rönicke et al., 2011), which can counteract early AD impairments not only for single neurons (Opazo et al., 2018; Rönicke et al., 2011; Dainauskas et al., 2023b), but also at a network level in memory storage and recall, as indicated by the results reported above in this work.

It is worth noting that, despite the relatively small number (100) of CA1 pyramidal neurons represented in our network, the underlying model for memory storage, originally developed by Cutsuridis et al. (2010), has been shown to capture the core functions of hippocampal processing and learning. In fact, the presence of all relevant types of inhibiting interneurons and the theta rhythm modulation of memory storage upon the gamma bursts coming from CA3 inputs reproduce the most important features of memory storage in hippocampus. Moreover, as shown above, 100 CA1 neurons are sufficient to explore the quality of memory storage both for orthogonal and non-orthogonal patterns, thus giving a complete overview of network performance in the various conditions investigated. In fact, the model has been used and extended for several applications and remains a valid tool in computational investigations of hippocampal encoding and retrieval; see, e.g., Bianchi et al. (2014), Andreakos et al. (2021, 2025), Cutsuridis (2012), Cutsuridis and Poirazi (2015), Cutsuridis (2019), Cutsuridis and Taxidis (2013) and Sutton and Ascoli (2021).

While the present study focuses exclusively on neuronal mechanisms underlying memory storage and recall, it is important to acknowledge the growing body of experimental evidence suggesting the role for astrocytes in these processes under physiological, non-pathological conditions. Astrocytes have been shown to modulate synaptic plasticity, for example via regulation of presynaptic and postsynaptic NMDAR function through gliotransmitters such as glutamate and D-serine, as well as through metabolic support mechanisms that contribute to long-term memory consolidation (Panatier et al., 2006; Henneberger et al., 2010; Suzuki et al., 2011). More recent *in vivo* studies further suggest that astrocyte populations may participate in shaping network activity during memory encoding and recall at the circuit level (Adamsky et al., 2018; Kol et al., 2020; Williamson et al., 2025; Dewa et al., 2025). In addition, experimental and computational modeling studies have proposed mechanistic roles for neuron-astrocyte interactions in spike-timing-dependent plasticity during development (Min and Nevian, 2012; Manninen et al., 2020). However, although astrocytes have been increasingly implicated in memory-related processes under physiological conditions, their precise roles remain debated, particularly when considering the transition from control to early-phase of disease states in hippocampus.

In this broader context, multiple mechanisms associated with synaptic dysfunction and, in some cases, impaired LTP and memory in AD have been investigated, including tau-related pathology (Kovacs, 2020), astrocyte–neuromodulator interactions, e.g., serotonin (Toivari et al., 2011), and broader glial signaling involving astrocytes and other cell types (Fernández-Matarrubia et al., 2025). In the present work, we focus on the relationship between accumulated Aβ and the GluN2B subunit, given its particular relevance to early functional changes that precede the cellular loss. Incorporating astrocytic dynamics and other molecular mechanisms into large-scale network models represents an important but non-trivial extension beyond the scope of the current study, which is intentionally restricted to well-established neuronal processes to establish a controlled baseline and framework for modeling memory storage and recall. Future work can incrementally integrate these additional mechanisms to further elucidate their contribution to network-level dysfunction; for examples, see Tesler et al. (2023) and Jiang et al. (2025) for network synchronization and neurovascular coupling.

In summary, we demonstrate that Aβ-induced synaptic impairments and their partial recovery via GluN2B modulation can be captured at the network level in a biologically grounded hippocampal model. By linking NMDAR-dependent synaptic plasticity to network-level dynamics of memory storage and recall, our results bridge the gap between cellular mechanisms and system-level cognitive function. Importantly, the model provides a mechanistic framework to study how early synaptic dysfunction propagates to network-level deficits and how targeted interventions can restore function. These findings highlight the value of integrating biophysical synaptic models into network simulations as a tool for exploring disease mechanisms and potential therapeutic strategies in Alzheimer’s disease.

## Data Availability

The raw data supporting the conclusions of this article will be made available by the authors, without undue reservation.
